# Scalable manufacturing of clinical‐grade differentiated cardiomyocytes derived from human‐induced pluripotent stem cells for regenerative therapy

**DOI:** 10.1111/cpr.13248

**Published:** 2022-05-09

**Authors:** Yuika Morita, Yoshikazu Kishino, Keiichi Fukuda, Shugo Tohyama

**Affiliations:** ^1^ Department of Cardiology Keio University School of Medicine Tokyo Japan

## Abstract

Basic research on human pluripotent stem cell (hPSC)‐derived cardiomyocytes (CMs) for cardiac regenerative therapy is one of the most active and complex fields to achieve this alternative to heart transplantation and requires the integration of medicine, science, and engineering. Mortality in patients with heart failure remains high worldwide. Although heart transplantation is the sole strategy for treating severe heart failure, the number of donors is limited. Therefore, hPSC‐derived CM (hPSC‐CM) transplantation is expected to replace heart transplantation. To achieve this goal, for basic research, various issues should be considered, including how to induce hPSC proliferation efficiently for cardiac differentiation, induce hPSC‐CMs, eliminate residual undifferentiated hPSCs and non‐CMs, and assess for the presence of residual undifferentiated hPSCs in vitro and in vivo. In this review, we discuss the current stage of resolving these issues and future directions for realizing hPSC‐based cardiac regenerative therapy.

## Graphical abstract

Heart disease is the leading cause of death worldwide. No cure for severe heart failure has been established other than heart transplantation, and the number of donors is insufficient. Therefore, hPSC‐derived CM (hPSC‐CM) transplantation is expected to replace heart transplantation. To prepare a large number of hPSC‐CMs more efficiently, an effective method for proliferating hPSCs while maintaining their undifferentiated state is needed. It is also desirable to induce large numbers of hPSC‐CMs at the same time and develop non‐invasive methods to prepare hPSC‐CMs only. Then. it is crucial to completely eliminate the undifferentiated hPSCs in advance to ensure a safe hiPSC‐CM transplantation for regenerative therapy. Moreover, assessment of the presence of residual undifferentiated hPSCs *in vitro* and *in vivo* should be considered. After evaluating the contamination of hPSCs *in vitro*, cardiac spheroids are generated for transplantation, and these spheroids are measured for contractility.

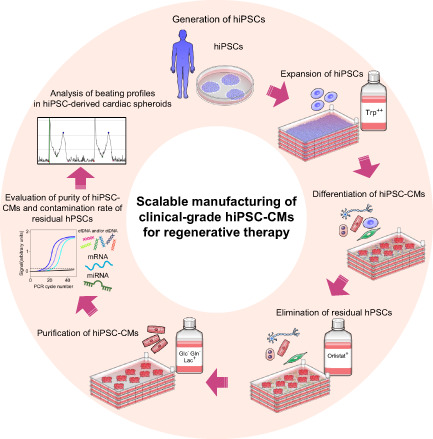

## 1 INTRODUCTION

Heart disease is the leading cause of death worldwide. No cure for severe heart failure has been established other than heart transplantation, and the number of donors is insufficient. To achieve an alternative to heart transplantation, the integration of medicine, science, and engineering is needed. Therefore, basic research aimed at cardiac regenerative therapy is currently one of the most active complex fields.

To date, a lot of basic research has provided numerous clues for the development of cardiac regenerative therapy, and the underlying treatment involves replacing damaged, necrotic, or fibrotic myocardial tissue with healthy myocardium. Given a large number of differentiated cardiomyocytes (CMs) required for clinical applications, it is ideal to use human pluripotent stem cells (hPSCs) that are self‐renewing and capable of differentiating any cell types of the three germ layers.

**FIGURE 1:**
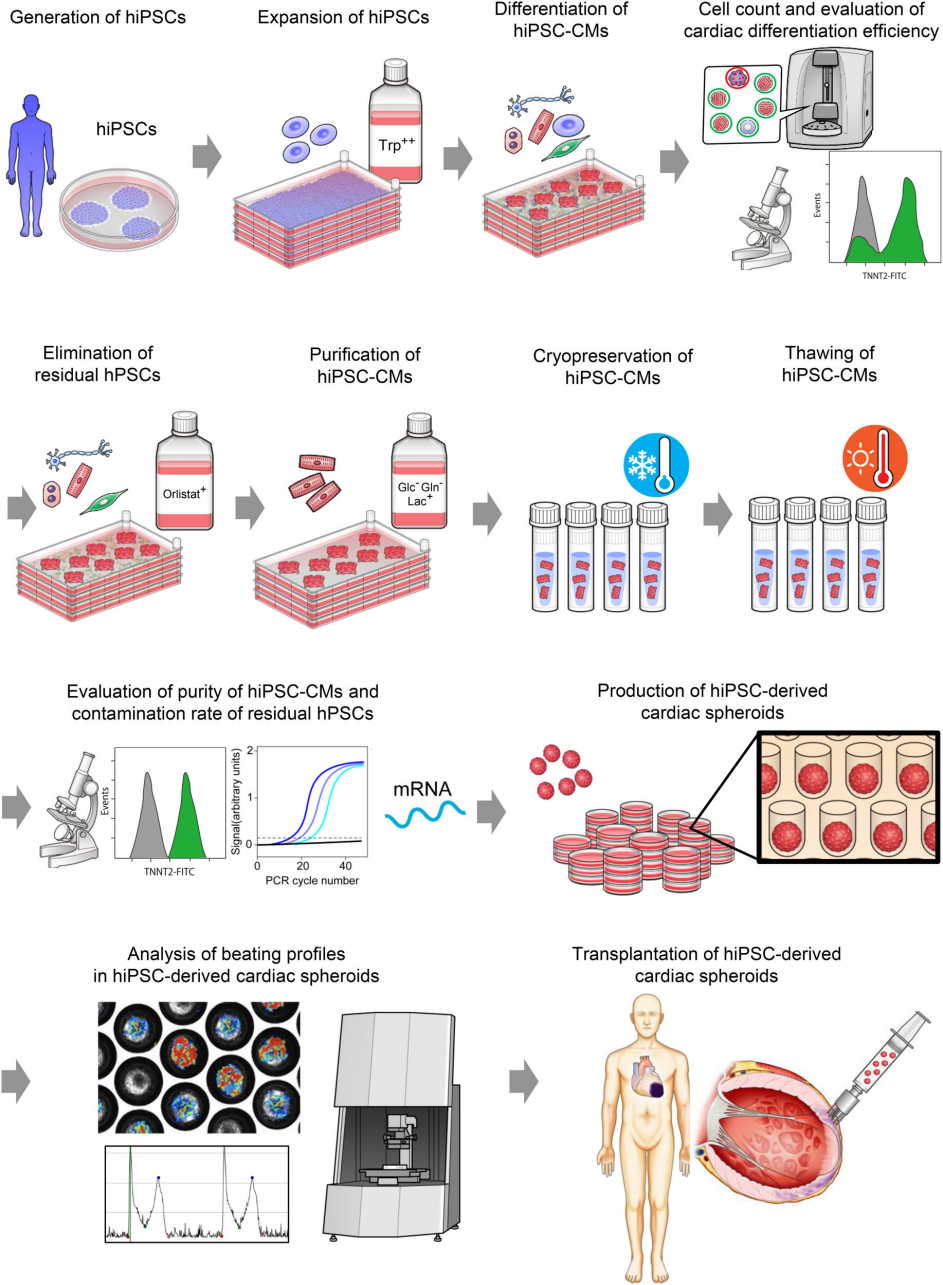
Scalable manufacturing of clinical‐grade hiPSC‐CMs. Tryptophan‐fortified media promotes the proliferation of hiPSCs. Large numbers of hiPSC‐CMs are induced in a multilayer culture plate. Cardiac differentiation efficiency is evaluated by cell count, flow cytometry, and immunostaining. Orlistat treatment selectively eliminates residual undifferentiated hPSCs. Then, hiPSC‐CMs were metabolically selected with glucose‐ and glutamine‐depleted lactate‐supplemented media. After purification, hiPSC‐CMs are isolated, harvested, and cryopreserved. The purity of hiPSC‐CMs and the contamination rate of residual undifferentiated hPSCs are assessed. After thawing, cardiac spheroids are produced. Beating profiles of cardiac spheroids are evaluated before transplantation. Cardiac spheroids are transplanted using our developed spheroids transplantation device.

Many methods for producing large numbers of differentiated CMs from hPSCs have been reported by utilizing the metabolic characteristics of hPSCs and differentiated CMs. To prepare a large number of hPSC‐derived CMs (hPSC‐CMs) more efficiently, an effective method for proliferating hPSCs while maintaining their undifferentiated state is needed. Methionine (Met), one of the essential amino acids, is highly consumed to maintain pluripotency, and the Met‐depleted medium induces endoderm differentiation. Recently, we reported that tryptophan supplementation promotes cell proliferation while maintaining pluripotency.^1^ In addition, non‐invasive methods for the purification of hPSC‐CMs and elimination of residual undifferentiated hPSCs are also required for successful and safe transplantation. We previously showed that glucose and glutamine are indispensable for the survival of non‐CMs, including residual undifferentiated hPSCs, and these depleted conditions enable the purification of hPSC‐CMs.^2^, ^3^, ^4^ In addition, it is necessary to establish a method to minimize the contamination of residual undifferentiated hPSCs. A major advantage of metabolic selection is that it can handle a large number of cells at once and does not require genetic modification. For instance, PluriSln, a small molecule that has screened out 52,000 candidates for eliminating hPSCs, specifically induces cell death, while progenitors and differentiated cells are sparing.^5^ In addition, *de novo* fatty acid (FA) synthesis is crucial for hPSC survival, and blockade of FA synthesis leads to mitochondrial‐mediated apoptosis of hPSCs.^6^, ^7^ These results suggest that metabolism‐based methods for proliferation and purification are very advantageous for application in regenerative medicine. To realize cardiac regenerative medicine, it is also necessary to evaluate the existence of residual undifferentiated hPSCs *in vitro* and *in vivo*. To assess the residual undifferentiated hPSCs *in vitro*, many methods have been reported, including flow cytometry, quantitative PCR (qPCR), droplet digital PCR (ddPCR), Raman spectrometry, ELISA, and biosensors. We reviewed the detection efficiency of residual undifferentiated hPSCs using these methods. Tumorigenicity studies to assess the risk of tumorigenesis when residual undifferentiated hPSCs are injected *in vivo* are also needed. Finally, we reviewed the clinical applications of hPSC‐based regenerative medicine (Figure 1).

## 2 GENERATION OF CLINICAL‐GRADE HIPSCS

Since Takahashi and Yamanaka first established human‐induced pluripotent stem cells (hiPSCs) in 2007, hiPSCs have held great promise for regenerative medicine. To accomplish great achievements, quality and safety must be ensured. Therefore, the European Medicines Agency and United States Food and Drug Administration (FDA) have defined Current Good Manufacturing Practice (cGMP) regulations. cGMP requires the manufacture and quality control of hiPSCs. Reagents used in hiPSC cultures affect the quality and safety of the cells because xeno reagents would not only increase the risk of infection but also immune rejection in hiPSC‐derived cell transplantation.^8^ In addition, tests against endotoxins and serious pathogenic microorganisms, such as mycoplasma and human immunodeficiency virus, are also needed to further ensure the safety for clinical applications.^9^ It has also been reported that clinical‐grade hiPSCs should meet the following requirements. First, parental cell donors must meet the guidelines for tissue donations. Second, the cell handling process must be performed in a GMP‐controlled environment using xeno‐free reagents. Third, clinical‐grade hiPSCs should be integration‐free and biologically safe.^10^ In addition, one of the most important aspects to avoid is the integration of external genes during reprograming of hiPSCs. Initially, hiPSCs were generated by retroviruses expressing *OCT4, SOX2, KLF4*, and *C‐MYC*. Random integration could cause insertional mutagenesis, and the possible though unlikely activation of oncogene *C‐MYC* may cause tumorigenesis. Therefore, it has previously been reported that Sendai virus, episomal vectors, minicircle DNA, mRNA, miRNA, and proteins are all integration‐free reprograming methods for hiPSCs.^11^, ^12^, ^13^, ^14^, ^15^, ^16^ In 2015, clinical‐grade hiPSCs were generated by integration‐free Sendai virus‐based reprograming under xeno‐free conditions.^10^ In addition, it also requires consistent operation of the whole process from the arrangements of biomaterial, culturing, and freezing of cells to quality control testing for the purpose of constantly manufacturing products of the same quality as the already established product specification. These processes are validated and executed according to the standard operating procedures (SOPs). Apart from SOPs, hiPSCs have some concerns owing to their characteristics.

First, hPSCs have often been observed to contain genomic mutations and abnormal karyotypes after passages.^17^, ^18^, ^19^ As hPSCs have low‐genomic stability, long culturing and frequent thawing of hPSCs affect karyotypic changes.^20^ Therefore, it is important to test them after extended passage and frequent freeze/thaw processes. Although the mechanism has not yet been elucidated, some chromosomes are commonly susceptible to genetic mutations, and some genetic aberrations have the advantage of proliferation, resistance to apoptosis, and differentiation propensity in hPSCs.^19^, ^21^, ^22^ Furthermore, it has been reported that cultured hESCs often have supernumerary centrosomes during mitosis, which is caused by overduplication within a single‐cell cycle and mitotic failure.^23^ This phenomenon is considered to be one of the causes of chromosome instability in cultured hESCs.

Second, epigenetic memories in somatic cells are inherited by hiPSCs. The epigenetic landscape, defined by histone and DNA modifications, is indispensable for maintaining cell identity. Rewriting epigenetic memories, that is, erasing epigenetic memories of somatic cells and replacing them with memories of hiPSCs, is an important aspect of inducing pluripotency. Kim et al. showed that low‐passage hiPSCs, which are reprogramed from adult somatic cells, have residual DNA methylation characteristics similar to those of somatic cells of origin, and this feature favors differentiation into cells of the somatic cell lineage of the donor.^24^ Another report showed that epigenetic memories are transient in early passage mouse iPSCs (miPSCs), and molecular and functional differences caused by epigenetic memories are lost through repeated passages.^25^ However, a subset of hiPSCs retains their epigenetic memory even after extended passaging.^26^, ^27^ From these results, genetic and epigenetic evaluation of hiPSCs is critical for their use in clinical applications.

For genetic testing of hiPSCs, karyotyping, G‐band analysis, qPCR, fluorescent *in situ* hybridization (FISH), microarray, whole‐genome/exome sequencing, and ddPCR are used. The advantages and disadvantages of these methods are listed in Table 1. Karyotyping is the most common strategy for detecting the size and structure of chromosomes. In standard G‐band analysis, it is necessary to analyze at least eight chromosomes and 20 metaphase counts.^28^

**TABLE 1:** Genetic stability assessment

	Method	Advantages	Disadvantages
Cells	Karyotyping	Whole‐genome analysis, Detection of aneuploidy, polyploidy, and other large chromosomal imbalances	Time consuming, Small resolution (High number of metaphases are needed), Cannot detect subkaryotypic variants
	FISH (fluorescent *in situ* hybridization)	Karyotype and information about mutations can be obtained	Probes must be known genes/mutations, Limited number of colors can be seen with fluorescent microscope, Not suitable for genome wide application
DNA	Microarray	Provide information on DNA regions with gains or losses	Cannot detect balanced rearrangements such as inversions
	Whole‐genome/exome sequencing	Very high and scalable throughput, High sensitivity and accuracy, Assess the whole genome at single‐base resolution	Expensive, Complex result interpretation
	PCR/ddPCR	High resolution for the CNV and SNV detection, Cost‐effective	Cannot comprehensive screening of chromosomal aberrations

Abbreviations: ddPCR, droplet digital PCR; CNV, copy number variation; SNV, single‐nucleotide variants.

qPCR is used to detect variants in regions that have already been reported. FISH relies on indirectly or directly labeled probes to detect specific target sequences with fluorescence in metaphase chromosomes. Although FISH has high sensitivity, it is only a detectable known genetic aberration and is not suitable for genome‐wide applications. ddPCR provides absolute quantification and detection of rare alleles independent of the number of amplification cycles when measuring the initial amount of nucleic acid in each sample, thus providing more precise and reproducible data than qPCR.^29^ It enables the detection of copy number variations and single‐nucleotide variants at a reasonable cost in comparison with next‐generation sequencing (NGS), FISH, and array comparative genomic hybridization. Although G‐band analysis, qPCR, and ddPCR are useful for analyzing known common genetic variants, these methods cannot detect variants if they are only present in 5–10% of the cells.^30^ Furthermore, genome mapping with long fluorescently labeled DNA molecules on nanochannel arrays was developed to detect whole‐genome structural variation.^31^ NGS has the advantage of analyzing the whole genome with high resolution and detecting most genomic abnormalities, but it is difficult to assess repeatedly due to cost, interpretation of complex results, and data analysis workload.^32^ Based on these observations, a method that is inexpensive, rapid, and accurate in its analysis is needed.

hiPSCs have two solid definitions: unlimited proliferative capacity and multiple differentiation ability. To produce a large number of hiPSC‐CMs for transplantation in patients suffering from severe heart failure, a large number of hiPSCs are also needed. In addition, the characteristics of hiPSC‐CMs vary slightly between experiments; therefore, it is preferable to induce a large number of hiPSC‐CMs at once. To achieve this, as it also requires a large amount of expensive media, it is desirable to develop a novel method to proliferate hiPSCs more efficiently and affordably without any changes in stemness or genetic mutation.

**FIGURE 2:**
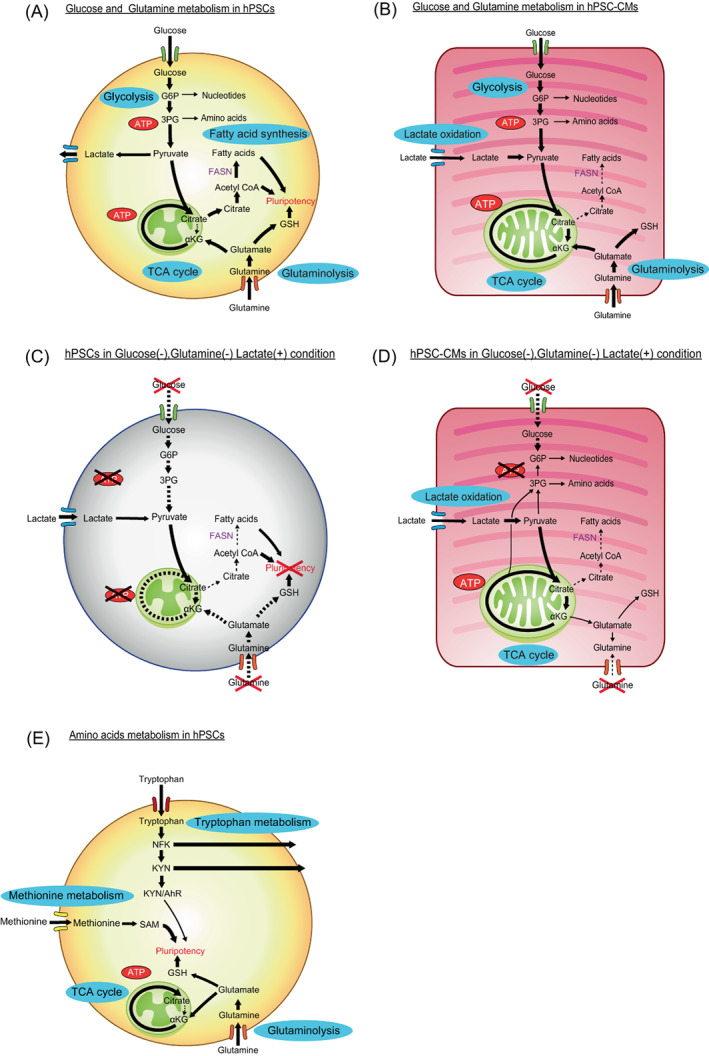
Overview of metabolic hallmarks in hPSCs and hPSC‐CMs. (A) hPSCs depend on glucose and glutamine metabolism for ATP and biomass production. They also show activated fatty acids synthesis for survival and maintenance of pluripotency. (B) hPSC‐CMs utilize glucose, glutamine, and lactate for ATP production via oxidative phosphorylation. (C) hPSCs cannot survive under glucose‐ and glutamine‐depleted with lactate‐supplemented conditions because they cannot utilize lactate efficiently. (D) hPSC‐CMs can survive under glucose‐ and glutamine‐depleted with lactate‐supplemented conditions because they can utilize lactate efficiently via oxidative phosphorylation. (E) Glutamine‐derived GSH, methionine‐derived SAM, and tryptophan‐derived KYN play key roles in the maintenance of pluripotency.

In this aspect, supplementation of biomass for cell proliferation, that is, the transition of metabolic pathways that are suitable for cell proliferation, is a feasible strategy. In particular, studies on cancer cell metabolism provide numerous insights into hPSC metabolism because cancer cells have a proliferative capacity as well as hPSCs, and these cells have a similar metabolic strategy. Many cancer cells depend on the excess uptake of glucose to survive, despite the cells being exposed to a high‐oxygen environment. This phenomenon, discovered by Otto Warburg, is called the Warburg effect or aerobic glycolysis and is the hallmark of all mammalian proliferating cells. hiPSCs are also highly dependent on glycolysis and secreted glucose‐derived lactate (Figure 2A).^2^ In cancer cells, abundant metabolites from aerobic glycolysis are utilized for glucose‐dependent lipid synthesis and non‐essential amino acid production. In addition to glucose, many cancer cell lines also prefer glutaminolysis. Glutaminolysis supplies nicotinamide adenine dinucleotide phosphate (NADP+), reduced (NADPH) required for multiple reactions: DNA synthesis, *de novo* FA synthesis, amino acid synthesis, and telomere maintenance.^33^ Thus, hPSCs are highly dependent on glucose and glutamine metabolism as a major energy source (Figure 2A, E).^2^, ^3^ As other metabolic pathways and enzymes for generating cytosolic NADPH, oxidative pentose phosphate pathway (oxPPP) branched from glycolysis, FA oxidation, folate‐mediated one‐carbon (1C) metabolism, malic enzyme 1 (ME1), isocitrate dehydrogenase 1 (IDH1), nicotinamide nucleotide transhydrogenase, and nicotinamide adenine dinucleotide (NAD) kinase have been recognized. The major suppliers of NADPH have been reported to be oxPPP, ME1, and 1C metabolism in proliferating and cancer cells.^34^, ^35^ A recent study revealed that oxPPP is a major producer of NADPH and loss of oxPPP by knockout of glucose‐6‐phosphate dehydrogenase (G6P) drastically decreases NADPH/NADP and cell growth in cancer cells.^36^ In addition, oxPPP dysfunction induces ME1 and IDH1 flux to generate NADPH from glutaminolysis, but folate‐mediated 1C metabolism cannot function because of dihydrofolate reductase dysfunction in G6P knockout cancer cells.^36^ These results suggest that oxPPP not only contributes to NAPDH generation but also folate‐mediated 1C metabolism. hPSCs also utilize glucose as a major energy source, and oxPPP‐related gene expression and metabolite levels are more abundant than hPSC‐CMs.^2^ With regard to the other amino acids for cancer proliferation, serine and glycine are well‐known and major carbon sources of folate‐mediated 1C metabolism. Serine is synthesized using 3‐phosphoglyceric acid and alpha‐ketoglutarate derived from glycolysis and glutaminolysis, respectively. 1C metabolism includes the folate and Met cycles; generates NADPH, nucleotides, and S‐adenosyl Met (SAM); and supports cell growth, proliferation, nucleotide synthesis, redox reductive metabolism, and DNA/histone methylation (Figure 2E).^37^ In hPSCs, serine and glycine are utilized to survive, but whether these amino acids are important for proliferation has not been analyzed. We previously evaluated the consumption profiles during the maintenance of hiPSCs and screened tryptophan as the most consumed amino acid. Under tryptophan‐fortified culture media (16‐fold tryptophan), the proliferation rate of hiPSCs was drastically increased compared with that of the control (Figure 2E).^1^ Immunohistochemistry results with NANOG, OCT4, SSEA4, and TRA‐1‐60 showed that there was no change in expression compared with the control. In addition, karyotype analysis showed no mutations in hiPSCs cultured with 16‐fold tryptophan‐fortified medium.^1^ Tryptophan‐fortified culture medium is one of the most suitable media for clinical application because the composition of the medium is clear, and hiPSCs proliferate in large quantities at low cost without causing gene mutations.

## 3 PRODUCTION OF CLINICAL‐GRADE HPSC‐CMS

It is estimated that more than 1 × 10^9^ hiPSC‐CMs per patient would be required to repair the loss of cardiomyocytes.^38^ To date, many methods have been reported for cardiac differentiation derived from hPSCs, and it has been suggested that these hPSC‐CMs show various characteristics because these methods have different points in the culture system 2D or 3D and combination of recombinant proteins and small molecules (Table 2).^4^, ^39^, ^40^, ^41^, ^42^, ^43^, ^44^, ^45^, ^46^, ^47^, ^48^, ^49^, ^50^, ^51^, ^52^, ^53^, ^54^ As there is a concern about a large number of variations in hPSC‐CMs for transplantation,^55^ it is desirable to induce large numbers of hPSC‐CMs at the same time and develop non‐invasive methods to prepare hPSC‐CMs only. One of the concerns with regard to mass culture in 2D culture is that there are fluctuations in differentiation efficiency, resulting from starting cell density and cell confluency.^48^, ^49^ In a 3D culture system using a bioreactor, there is no need to consider these points, although attention should be paid to the variability in the size of individual spheroids. However, once the hPSC‐CMs are aggregated, it is difficult to completely dissociate into single cells, and cell aggregates are removed by a cell strainer,^56^ which may result in cell loss. To obtain a large number of hiPSC‐CMs in 2D culture, we developed a method to induce hiPSC‐CMs in a multilayer culture plate with active gas ventilation.^4^ In this system, hiPSCs were efficiently differentiated into CMs, and these cells started beating on day 7–10. Cell viability was sustained at over 95% and the total number of hiPSC‐CMs in single, 4, and 10 layers under active gas ventilation was 1.5 × 10^8^, 6.7 × 10^8^, and 1.5 × 10^9^ cells, respectively. We previously showed that hiPSC‐derived non‐CMs highly depend on glycolysis and glutaminolysis.^2^, ^3^ These cells cannot survive under glucose‐ and glutamine‐depleted conditions (Figure 2C). Furthermore, hPSCs hardly uptake lactate and convert lactate to pyruvate, thus, it is difficult for hPSCs to use lactate as an energy source. In contrast, hiPSC‐CMs uptake lactate which is incorporated as pyruvate in the tricarboxylic acid (TCA) cycle (Figure 2B,D).^3^ These metabolic differences enable the purification of >99% hiPSC‐CMs.^2^, ^3^ Applying this method to differentiation in multilayer culture plates, hPSC‐CMs were metabolically selected with glucose‐ and glutamine‐depleted lactate‐supplemented media, and over 1.0 × 10^9^ hiPSC‐CMs were obtained in 10‐layer culture plates at the same time after purification, and immunocytochemistry data showed that almost all cells were cTNT positive.^4^ Interestingly, hiPSC‐CMs cultured in glucose‐depleted lactate‐supplemented conditions showed a metabolically and transcriptionally mature direction.^57^, ^58^ After purifying hPSC‐CMs, it is desirable to preserve them as freeze stocks to save costs and allow long‐distance and term transportation. Therefore, metabolically selected cells were isolated and cryopreserved. After thawing, the viability of the cryopreserved cells was over 80%.^4^

**TABLE 2:** Cardiac differentiation method

Year of publication	Publication	Differentiation method	Culture media	Mesoderm induction	Cardiac specification	Differentiation efficiency	Reference
2001	Kehat et al.	3D	KO DMEM+20%FBS	NA	NA	8.1% beating EBs	^39^
2007	Laflamme et al.	2D	RPMI1640 + B27	Activin A, BMP4	NA	>30% β‐MHC(+) cells	^40^
2008	Yang et al.	3D	StemPro34	Activin A, BMP4, bFGF	VEGF, DKK1, bFGF	40%–50% TNNT2(+) cells	^41^
2009	Tran et al.	3D	KO DMEM+15%FBS	WNT3A	NA	50% α‐Actinin (+) cells (within beating clusters)	^42^
2011	Elliot et al.	2D/3D	LI‐APEL	Activin A, BMP4, bFGFVEGF, SCF, WNT3A	NA	3D: 38% NKX2‐5(+) cells 2D: 24% NKX2‐5(+) cells	^43^
2011	Kattman et al.	3D	StemPro34	Activin A, BMP4, bFGF	VEGF, DKK1, TGF*β*i, BMPi	>60% TNNT2(+) cells	^44^
2011	Zhang et al.	2D	RPMI1640 + B27	Activin A, BMP4, bFGF	NOGGIN, RA/RAi, DKK1	RA: 50.7 ± 1.7% TNNT2(+) cells RAi: 64.7% ± 0.9% TNNT2(+) cells	^45^
2011	Willems et al.	3D	StemPro34	Activin A, BMP4, bFGF	bFGF, IWR1, Triiodothyronine	30% α‐MHC(+) cells	^46^
2012	Zhang et al.	2D	RPMI1640 + B27 insulin(−)	Activin A, BMP4, bFGF	NA	40%–92% TNNT2(+) cells	^47^
2013	Lian et al.	2D	RPMI1640 + B27 insulin(−)	CHIR99021	IWP2	85% TNNT2(+) cells	^48^
2014	Burridge et al.	2D	CDM3	CHIR99021	WNT‐C59	80%–95% TNNT2(+) cells	^49^
2015	Devalla et al.	3D	BPEL	Activin A, BMP4, CHIR99021 SCF, VEGF	RA	50% NKX2‐5(+) cells	^50^
2017	Protze et al.	3D	StemPro34	Activin A, BMP4, bFGF	IWP2, VEGF, BMP4RA, bFGFi, TGFβi	5% NKX2‐5(+) cells 55% NKX2‐5(−) cells	^51^
2017	Tohyama et al.	2D	RPMI1640 + B27 insulin(−)	CHIR99021, BMP4	IWR1	80% TNNT2(+) cells	^4^
2017	Palpant e al.	2D	RPMI1640 + B27 insulin(−)	Activin A, BMP4, CHIR99021	XAV‐939	80% TNNT2(+) cells	^52^
2020	Laco et al.	3D	RPMI1640 + B27 insulin(−) + L‐ascorbic acid 2‐phosphate	CHIR99021	IWR1	68% TNNT2(+) cells	^53^

Abbreviations: bFGF; basic fibroblast growth factor, bFGFi; bFGF inhibitor, BMP; bone morphologic protein, BMPi; BMP inhibitor, CMs; cardiomyocytes, DKK; dickkopf, DMSO dimethyl sulfoxide, EB; embroid body, FBS; fetal bovine serum, KO; knock out, MLC; myosin light chain, NA; not applicable or not available, TGFβ; transforming growth factor β, TGFβi; TGFβ inhibitor, TNNT; troponin T, SCF; stem cell factor, VEGF; vascular endothelial growth factor, RA; retinoic acid, RAi; RA inhibitor, IWP; inhibitor of Wnt production.

## 4 ELIMINATION OF RESIDUAL UNDIFFERENTIATED HPSCS

Although it is ideal to induce all hiPSCs into hiPSC‐CMs with a cardiac differentiation method, it is currently difficult to establish such a method. Undifferentiated hPSCs can be a source of teratomas when transplanted, even if 0.025% of residual undifferentiated hPSCs remain in the differentiated cells,^59^ thus, it is crucial to completely eliminate the residual undifferentiated hPSCs in advance to ensure safe hiPSC‐CM transplantation for regenerative therapy. To address this issue, numerous strategies have been reported for genetic, biophysical, biochemical, and immunogenic approaches (Table 3).^2^, ^3^, ^5^, ^37^, ^60^, ^61^, ^62^, ^63^, ^64^, ^65^, ^66^, ^67^, ^68^, ^69^, ^70^, ^71^, ^72^, ^73^, ^74^, ^75^ Genetic approaches typically knock in an inducible suicide gene in the promoter region or gene body, which is expressed only in undifferentiated hPSCs.^65^, ^73^, ^75^ For example, it has been reported that killer red protein, which strongly induces phototoxicity by generating reactive oxygen species, induces the selective cell death of hPSCs.^65^ Furthermore, to improve the safety of hPSC‐based cell transplantation, hPSC lines with two drug‐induced safeguards that have different functions and address different safety concerns have also been reported.^75^ That is, one small molecule helps to eliminate residual undifferentiated hPSCs and the other small molecule helps to kill all of the hPSC‐derivatives in case adverse events arise *in vivo*. In addition, strategies for genetically and pharmacologically deleting survivin to induce apoptosis in hESCs and teratomas have also been reported.^74^ However, these strategies have a risk of off‐target effects during gene modification and increase the hurdle to using these cells in clinical applications. Using a biochemical approach, Yi et al. discovered the phosphor peptide D‐3 and showed that it can selectively and effectively eliminate residual undifferentiated hPSCs within just 1–2 h by binding to alkaline phosphatase on the surface of hPSCs.^67^ Moreover, it has also been reported that salicylic diamines, which inhibit mitochondrial ATP production by decreasing the oxygen consumption rate, show selective cytotoxicity to miPSCs and hiPSCs but not to miPSC‐CMs.^70^ However, salicylic diamines do not have any effect on miPSC‐CMs, and the exposure time allows selective elimination of miPSCs and hiPSCs. In addition, the elimination of miPSCs and hiPSCs by salicylic diamines is not complete and transplantation is compromised.^70^ Among these methods, metabolism‐based approaches offer some advantages in terms of cost, time, simplicity, scalability, and safety because they do not require gene modification, expensive antibodies, and time to perform cell sorting. To successfully eliminate residual undifferentiated hPSCs, it is important to understand the metabolic characteristics of hPSCs. As mentioned above, hPSCs are mainly dependent on glycolysis and oxPPP for ATP production and proliferation, thus, they cannot survive under glucose‐depleted conditions (Figure 2C).^2^ In addition, glutamine is a key metabolite for the survival of hPSCs. Glutamine is utilized in some contexts as a source of nucleotide synthesis, glutathione synthesis, non‐essential amino acid synthesis, and epigenetic modification. In cancer cells, glutamine contributes to FA synthesis via reductive carboxylation under hypoxia.^76^ We have previously shown that glutamine not only contributes to nucleotide and glutathione syntheses, but also ATP synthesis via the latter steps of the TCA cycle, and this unique pathway is indispensable for survival in hPSCs.^3^

**TABLE 3:** Elimination of residual hPSCs

Publication	Strategy	Residual cells	Method	Reference
Tohyama et al.	Metabolic	mESCs, hPSCs	Glucose‐depleted culture medium with abundant lactate	^2^
Tohyama et al.	Metabolic	hPSCs	Glucose‐ and glutamine‐depleted culture medium with abundant lactate	^3^
Ben‐David et al.	Metabolic	hPSCs	Oleate synthesis inhibition	^5^
Tanosaki et al.	Metabolic	hPSCs	Fatty acid synthesis inhibition	^7^
Shiraki et al.	Metabolic	hPSCs	Methionine‐free culture medium	^37^
Ben‐David et al.	Immunological	hPSCs	Claudin 6‐targeted selection	^60^
Okada et al.	Immunological	hPSCs	GPC3 reactive cytotoxic T lymphocyte‐based selection	^61^
Sougawa et al.	Immunological	hiPSCs	Brentuximab vedotin inducible cytotoxicity	^62^
Schriebl et al.	Immunological	hESCs	hESC‐specific antibodies based selection	^63^
Nagashima et al.	Biophysical	hiPSCs	Treatment with high concentration of L‐alanine	^64^
Cho et al.	Biophysical	mESCs (Insertion of suicide gene)	Phototoxicity	^65^
Kim et al.	Biochemical	hiPSCs	BV treatment	^66^
Kuang et al.	Biochemical	hPSCs	D‐3 treatment	^67^
Matsumoto et al.	Biochemical	hiPSCs	Plasma‐activated medium induced selective cell death	^68^
Tateno et al.	Biochemical	hPSCs	Lectin‐toxin fusion protein	^69^
Burkert et al.	Biochemical	hPSCs	Salicylic diamine treatment	^70^
Wu et al.	Genetic	hPSCs (In‐frame *iC9* gene insertion into the *SOX2* locus to target undifferentiated hESCs)	Induction of apoptosis by *iC9* inducer AP1903	^71^
Elovic et al.	Genetic	hESCs (miR‐499‐responsive lethal mRNA is designed)	Induction of apoptosis by miR‐499‐responsive lethal mRNA	^72^
Parr et al.	Genetic	hPSCs (miRNA switch [miR‐302/367] is encoded)	Puromycin selection	^73^
Blum et al.	Genetic	hESCs (A plasmid containing the dominant negative survivin isoform fused in frame to GFP is transfected)	Genetic and pharmacological ablation of survivin	^74^
Martin et al.	Genetic	hPSCs (*NANOG* ^*iC9‐YFP*^ and *ACTB* ^*OiC9‐mPlum*^ */ACTB* ^*TK‐mPlum*^ are introduced)	Drug inducible selection	^75^

Abbreviations: BV, Ban venom; GPC3, Glypican‐3; iC9, Inducible caspase‐9; OiC9, Orthogonal inducible caspase‐9; TK, thymidine kinase.

Similar to other amino acids, mESCs are dependent on threonine catabolism, which regulates intracellular SAM production. Knockdown of threonine dehydrogenase (*Tdh*) decreases the accumulation of SAM and trimethylation of histone H3 lysine 4 (H3K4me3), leading to slow growth and increased differentiation.^77^, ^78^ However, human *TDH* is a nonfunctional pseudogene, which suggests the possibility that hPSCs have other functions to maintain the level of SAM for pluripotency. Shiraki et al. showed that Met metabolism is crucial for hPSC survival (Figure 2E).^37^ Met depletion induces upregulation of the p53‐p38 signaling pathway, which is critical for cell cycle arrest and survival.^37^ In the short‐term depletion of Met, the abbreviated G1 phase, which is characteristic of hPSCs, is prolonged and finally leads to cell cycle arrest and causes the differentiation of three germ layers due to histone and DNA methylation and a decrease in *NANOG* expression. p53 binds to the *NANOG* promoter and negatively regulates mESCs.^79^ However, long‐term depletion of Met is useful for removing hPSCs, and long‐term exposure may affect the desired cells because Met is an essential amino acid and one of the critical 1C metabolism units to synthesize polyamine, nucleotides, and glutathione, as well as being a supplier for epigenetic modification. In relation to 1C metabolism, it has been reported that the knockout of Lin28A and Lin28B, which are RNA‐binding proteins in mPSCs, exacerbated glucose incorporation into serine. As a result, nucleosides and nucleotides were downregulated, leading to a decrease in the number of mPSCs. In contrast, supplementing culture media with nucleotides rescues proliferation defects in mPSCs, suggesting that glucose‐derived serine synthesis regulated by Lin28 is indispensable for the survival of mPSCs to supply nucleotides.^80^

Cancer cells share many metabolic features with PSCs; in particular, the multiple roles of FA metabolism have been reported more in cancer cells than in PSCs. *De novo* FA synthesis is an essential cellular program that converts nutrients into metabolic intermediates for energy storage, membrane components, and signaling molecules for cancer cell growth and survival.^81^ With regard to miPSCs, *de novo* FA synthesis regulates cellular reprograming and pluripotency through mitochondrial fission.^6^ Overexpression of Acc1, the rate‐limiting enzyme of *de novo* FA synthesis, promotes cellular triglyceride levels in MEF and mitochondrial fission, and markedly increases miPSC colonies. The mechanism by which Acc1 regulates FA synthesis in mPSCs has two scenarios: consumption of AcCoA, which affects acetylated mediated Fis1 ubiquitin‐proteasome degradation, and generation of the lipid component of mitochondria toward fission. In addition to mPSCs, we demonstrated that undifferentiated hPSCs are highly dependent on FA synthesis compared with hPSC‐CMs (Figure 2A).^7^ Detailed lipid profiling has revealed that FA synthase (FASN) inhibition decreases sphingolipid and phosphatidylcholine (PC) levels. Notably, PC is especially important for hPSCs to survive, and hPSCs with FASN inhibition are rescued by PC supplementation. In addition, transplanted cells treated with orlistat did not show complete teratoma formation.^7^ This result shows that it is useful to selectively eliminate residual undifferentiated hPSCs in differentiated cells for the prevention of tumor formation in regenerative therapy. Moreover, orlistat has already been approved by the FDA as an anti‐obesity drug. To date, as there are many inhibitors of FA synthesis, it is interesting to note that these inhibitors are effective in eliminating residual undifferentiated hPSCs, such as orlistat, and there are differences in their mechanism and dosage. For the elimination of undifferentiated mPSCs, doxorubicin, which belongs to the class of anthracyclines often used in combination with other medications to treat some cancers, is used.^82^, ^83^ Although doxorubicin is also approved by the FDA as well as orlistat, a high dose of doxorubicin can increase the risk of congestive heart failure.^84^ Chour et al. showed that low‐dose doxorubicin is effective in eliminating proliferative hESCs from differentiated hESC‐CMs.^85^ Low‐dosage doxorubicin administration did not affect the gene expression and proteome profiles of hESC‐CMs.^85^ In the teratoma formation assay, doxorubicin‐treated cells were not proliferative, and no teratomas were observed in vivo. Nevertheless, the role of apoptosis in doxorubicin‐induced cardiotoxicity has been well established, and it seems unsuitable for the selective elimination of residual undifferentiated hPSCs in hiPSC‐CMs.^86^ In addition, using brentuximab vedotin, which is effective in eliminating CD30‐positive cancer cells, hPSCs have also been used to eliminate residual undifferentiated hPSCs because they also express CD30.^62^

## 5 *IN VITRO* TUMORIGENICITY TESTS FOR HPSC‐DERIVED PRODUCTS

For clinical applications, it is crucial to establish a system to eliminate residual undifferentiated hPSCs and for assessing the contamination of undifferentiated hPSCs. Many methods for the evaluation of residual undifferentiated hPSCs have been developed, but internationally accepted standard methods have not yet been established as of 2018.^87^ However, there is increasing recognition that the assessment of residual undifferentiated hPSCs is indispensable for the safe transplantation of hPSC derivatives. Therefore, it is important to combine methods (i.e., flow cytometry, qPCR, ddPCR, Raman spectrometry, immunocytochemistry, ELISA) considering the advantages and disadvantages of these methods.

As mentioned above, 0.025% contamination of residual undifferentiated hPSCs can be a risk factor for tumorigenesis in the transplantation of hPSC derivatives.^59^ Therefore, it is crucial to develop or select highly quantitative methods and suitable factors for detection. The detection methods and efficiency are listed in Table 4.^88^, ^89^, ^90^, ^91^, ^92^, ^93^, ^94^, ^95^ Flow cytometry is one of the most common strategies for the detection of residual undifferentiated hPSCs using antibodies against hPSC‐specific transcription factors and/or cell surface markers. Kuroda et al. assessed the detection efficiency of hPSCs using five antibodies that recognize stem cell marker antigens (OCT4, NANOG, SOX2, TRA‐1‐60, and TRA‐1‐81) and showed that anti‐OCT4, anti‐SOX2, and anti‐TRA‐1‐60 antibodies distinguished hiPSCs from hiPSC‐derived retinal pigment epithelial cells (hiPSC‐RPE).^88^ In addition, residual undifferentiated hPSCs were identified as TRA‐1‐60‐positive cells in 0.1% and 0.01% spiked samples via flow cytometry.^88^ The surface‐enhanced Raman scattering‐based assay showed that the detection limit of SSEA‐5 and TRA‐1‐60 was 0.0001%, and the detection efficiency was improved dramatically.^89^ These results suggest that adequate selection of hPSC‐specific markers and highly sensitive methods is both necessary to accomplish the successful detection of residual undifferentiated hPSCs. Although flow cytometry has some advantages in terms of speed and quantification, it should be noted that the gating technique highly affects these results.

**TABLE 4:** In vitro tumorigenicity test

Publication	Method	Markers	Residual cells	Differentiated cells	Detection rate	Reference
Kuroda et al.	Flow cytometry	OCT4, NANOG, SOX2, TRA‐1‐60, TRA‐1‐81, TRA‐2‐49	hiPSCs	RPE cells	0.01–0.1% (TRA‐1‐60)	^88^
Han et al.	Raman Spectroscopy	SSEA‐5 conjugated nanoparticle,TRA‐1‐60 conjugated nanoparticle	hiPSCs	NIH3T3 cells	0.001%	^89^
Han et al.	Flow cytometry	SSEA‐5	hiPSCs	NIH3T3 cells	0.1%–1.0%	^89^
Ito et al.	Flow cytometry	TRA‐1‐60	hiPSCs	hiPSC‐CMs	0.1%	^90^
Kuroda et al.	Soft agar		PA1	RPE cells	1%	^88^
Tano et al.	Soft agar		hiPSCs	Neurons or hMSCs	0.001%–0.01%	^92^
Tateno et al.	ELISA	TRA‐1‐60 epitope, TRA‐1‐80 epitope	hiPSCs	hNSCs	0.05%	^93^
Kuroda et al.	qRT‐PCR	*TRA‐1‐60, OCT4, KLF4*, *c‐MYC, SOX2, NANOG*, *LIN28, REX1*	hiPSCs	RPE cells	0.01% (*LIN28*)	^88^
Ito et al.	qRT‐PCR	*LIN28*	hiPSCs	Primary CMs	0.001%	^90^
Sekine et al.	qRT‐PCR	*ESRG, CNMD,SFRP*	hiPSCs	HE cells	0.005% (*ESRG*). 0.025% (*CNMD*, *SFRP*)	^91^
Artyuhov et al.	qRT‐PCR	*TGDF1*	hiPSCs	NPCs and dermal fibroblasts	0.01%	^94^
Artyuhov et al.	ddPCR	*TGDF1*	hiPSCs	NPCs	0.002%	^94^
Kuroda et al.	ddPCR	*LIN28*	hiPSCs	hiPSC‐CMs	0.001%	^95^

Abbreviations: RPE, retinal pigment epithelium; HE, hepatic endoderm; MSC, mesenchymal stem cell; NSC, neural stem cell; NPC, neural progenitor cell; CM, cardiomyocyte.

qPCR is simple and fast, but has the disadvantage that it is difficult to determine the exact number of residual undifferentiated hPSCs. A spike assay with hPSCs in hepatic endoderm showed that the detection limits for *ESRG* (*Embryonic Stem Cell Related*), *CNMD* (*Chondromodulin*), and *SFRP* (*Secreted Frizzled Related Protein 2*) were 0.005%, 0.025%, and 0.025%, respectively, while those for *SOX2*, *OCT4*, and *NANOG* were 1%, 2.5%, and 5%, respectively.^91^ *LIN28* was not suitable for the detection of hPSCs in the hepatic endoderm because the detection limit of *LIN28* was 5%.^91^ Other reports showed that the detection limits for *SSEA‐5* and *TRA‐1‐60* were 0.1%–1.0% and 0.01%–0.1%, respectively.^88^, ^89^, ^90^ ddPCR enables direct quantification of DNA/mRNA copies, and it can prevent the bias that comes from non‐target gene amplification using qPCR. Furthermore, it enriches the template in partitioning and enables more sensitive detection of rare targets than PCR.^96^ It is also used to quantify circulating fetal and maternal DNA from cell‐free plasma.^96^ ddPCR analysis with LIN28 probes detected as low as 0.001% residual undifferentiated hPSCs in primary CMs. Artyuhov et al. showed that the detection limit of *OCT4*, *TGDF*, and *LIN28* was 0.01%, whereas that with ddPCR was 0.002%.^94^ Watanabe et al. reported a detection efficiency of 0.00002% when ddPCR was performed after enrichment of stem cell markers using magnetic beads.^97^ In addition, it has been reported that the detection efficiency of hPSCs in hPSC‐CMs suggests that ddPCR is more suitable than qPCR for the assessment of residual undifferentiated hPSCs.

## 6 IN VIVO TUMORIGENICITY TESTS FOR HPSC‐DERIVED PRODUCTS

Monitoring hPSC‐CMs after transplantation is important, even if it is confirmed that there are no undifferentiated hPSCs remaining before transplantation. The general approach for tumorigenicity testing is based on ectopic transplantation into small animals, but the detection efficacy of tumors varies depending on the animal strain and immunosuppression. Therefore, the test animals should be deficient in cytotoxic T‐lymphocyte activity.^98^To investigate the safety of transplanted cells, undifferentiated hPSCs and tumor cell lines were transplanted and the duration of tumorigenesis examined (Table 5).^59^, ^99^, ^100^, ^101^, ^102^, ^103^ Some reports state that non‐obese diabetic (NOD)/severe combined immunodeficiency (SCID)‐IL2Rγ^null^ (NOG or NSG) mice are more suitable for testing for tumorigenesis because these mice are defective in T, B, and natural killer cells and show high efficiency with engraftment of human cells and tissues compared with conventional T cell‐defective nude mice.^104^, ^105^ It has been reported that tumor‐producing doses (TPD_50_) of HeLa cells are 1.3 × 10^4^ and 4.0 × 10^5^ cells in NOG and nude mice, respectively, after 16 weeks of inoculation.^99^ However, it is often preferable to use large animals because it must be administered in the same amount as the patient would be given, or in the maximum feasible dose that can be implemented. In addition, small animals have a short lifespan. NOD/SCID mice develop thymic lymphomas frequently with age.^106^, ^107^ This incidence often results in shortened life span, which can confound the results of tumorigenicity testing.^99^ The transplantation method needs to be considered. hPSCs are generally injected as clumps because hPSCs easily induce apoptosis in a single‐cell state. Therefore, it is difficult to transplant accurate cell numbers into animals. To resolve this issue, it has been reported that a mixture of hPSCs as a single state and mitomycin‐C‐treated fibroblasts with Matrigel could be engrafted into SCID and NOD/SCID mice.^59^, ^102^ It has been reported that the tumor‐formation capacity of hPSCs in NOG mice depends on the method of transplantation. In this study, tumor formation was monitored for 16 weeks and showed the TPD_50_ of three patterns: hiPSC clumps, single hiPSCs/neonatal human dermal fibroblasts (NHDFs), and single hiPSCs/NHDFs supplemented with a ROCK inhibitor, which were 681, 4632, and 631 cells, respectively. This result suggests that a ROCK inhibitor drastically improved transplantation efficiency.^103^ Previous studies have evaluated the differences in the tumorigenic potential of different 10 hiPSC lines, and 3.0 × 10^4^ cells of single cell‐dissociated hiPSCs were injected at the subcutaneous sites of NOG mice with 1.0 × 10^6^ cells of mitomycin‐C‐treated NHDFs with Matrigel and a ROCK inhibitor. After 16 weeks of monitoring, it was revealed that the 10 hiPSC lines differed in tumor incidence, formation latency, and volume, indicating a variety of tumorigenicity in these hiPSC lines. Almost all teratomas were categorized as immature teratomas and did not show any symptoms of carcinoma or sarcoma.^103^

**TABLE 5:** *In vivo* tumorigenicity test

Publication	Transplanted cells	TPD50	Matrigel	Transplanted animals	Reference
Kusakawa et al.	HeLa cells	1.3 × 10^4^ cells (at 16 weeks)	−	NOG mice	^99^
	HeLa cells	4.0 × 10^5^ cells (at 16 weeks)	−	nude mice	
	HeLa cells	7.9 × 10 cells (at 16 weeks)	+	NOG mice	
Kanemura et al.	HeLa cells	3.5 (Log10) (at 8 weeks)	+	nude mice	^100^
	HeLa cells	4.9 (Log10) (at 10 weeks)	−	nude mice	
	HeLa cells	2.5 (Log10) (at 11 weeks)	+	SCID mice	
	HeLa cells	3.83 (Log10) (at 11 weeks)	−	SCID mice	
	HeLa cells	2.17 (Log10) (at 16 weeks)	+	NOD‐SCID mice	
	HeLa cells	3.5 (Log10) (at 14 weeks)	−	NOD‐SCID mice	
	HeLa cells	1.1 (Log10) (at 18 weeks)	+	NOG mice	
	HeLa cells	3.97 (Log10) (at 13 weeks)	−	NOG mice	
	HeLa cells	1.32 (Log10) (at 33 weeks)	−	nude rats	
	hiPSCs	4.73 (Log10) (at 33 weeks)	−	nude rats	
Yasuda et al.	hiPSC clumps	6.8 × 10^2^ cells (at 16 weeks)	−	NOG mice	^103^
	Single hiPSCs and NHDF	4.6 × 10^3^ cells (at 16 weeks)	−	NOG mice	
	Single hiPSCs and NHDF+ROCK inhibitor	6.3 × 10^2^ cells (at 16 weeks)	−	NOG mice	

Abbreviations: NHDF, Normal human neonatal dermal fibroblasts; TPD50, tumor‐producing dose at the 50% endpoint.

It is necessary to observe for a certain period that hPSC‐derived cells are safe and that tumors do not form when hPSC‐derived target cells are transplanted. Transplantation experiments of hPSC‐RPE cells for age‐related macular degeneration are the most advantageous areas in the field of regenerative therapy using hPSCs. hPSC‐RPE cells were also transplanted into the monkey retinal degeneration model, and no tumor formation was observed after 4 months of transplantation.^108^ Preventing rejection of hPSC‐derived target cells in recipients is one of the greatest challenges in transplantation, and it is thought to be desirable that human leukocyte antigen‐haplotypes (HLA) are homologous between donors and recipients. This is commonly referred to as the major histocompatibility complex (MHC). Allogenic transplantation of MHC homozygote monkey iPSC‐RPE cells into the sub‐retinal space of MHC‐matched monkeys was also performed, which showed that there was no immunosuppression or tumorigenicity after 6 months of transplantation.^109^ HLA homozygote hPSC‐RPE cells have already been applied to five HLA‐matched patients, and all five of the cases were stable with no abnormal graft growth during the 1‐year observation period.^110^ In addition to the treatment of age‐related macular degeneration, clinical trials in retinitis pigmentosa, limbal stem cell deficiency, Parkinson's disease, spinal cord injuries, and heart failure are currently underway in Japan. In the field of Parkinson's disease research, hPSC‐dopaminergic neurons were transplanted into the brains of Parkinson's disease model monkeys, which showed behavioral improvement for at least 12 months, and the graft showed no malignant or teratomatous findings.^111^ As a simulation of a clinical trial, approximately 5 × 10^6^ cells were transplanted into the bilateral putamen of the Parkinson's disease model monkeys, and the monkeys were followed up for 2 years.^112^ Historical analysis showed that more than 1 × 10^5^ dopaminergic neurons survived per monkey, and there was no tumor formation.^112^ Furthermore, using hPSCs that were established from a healthy individual with the most common HLA haplotype in the Japanese population, hPSC‐dopaminergic progenitors were transplanted into the striatum of NOG mice and observed for 52 weeks.^113^ In this experiment, no graft‐related toxicity or distant metastasis to other organs was observed.^113^

## 7 EVALUATION OF CARDIAC FUNCTION AFTER TRANSPLANTATION OF HPSC‐CMS

In the field of cardiovascular disease research, it has been reported that intramyocardial direct injection of ~750 × 10^6^ hESC‐CMs was performed in monkeys with large myocardial infarction, and cardiac function was enhanced.^114^ However, post‐transplant ventricular arrhythmia was observed. In the case of MHC‐matched allogeneic transplantation of monkey‐PSC‐CMs, cardiac function was recovered, but post‐transplant ventricular arrhythmia was also observed.^115^ It has been considered that ventricular arrhythmia is caused by automaticity of hPSC‐CMs.^114^, ^116^ In the clinical application of human heart regeneration, cell sheets generated by autologous skeletal muscles were transplanted to the hearts of patients.^117^ It has been mentioned that this approach is feasible for the treatment of cardiomyopathy. However, some argue that demonstrating the effectiveness of the skeletal muscle transplantation as needed remains questionable. It has been reported that injecting skeletal myoblasts from the leg does not improve heart function.^118^ Therefore, cardiac regeneration using hPSC‐CMs is desirable. Menasche et al. implanted patches with hESC‐cardiac progenitors into patients and evaluated their efficacy at 1, 3, 6, and 12 months postoperatively.^119^ During follow‐up, no tumor was detected, and none of the patients presented with arrhythmia. All patients symptomatically improved with increased systolic motion of the cell‐treated segments. From these results, hiPSC‐derivatives were proven to be effective in treating diseases in patients. In the future, it is hoped that effective methods will become widespread and that many patients can be treated.

## 8 CONCLUSION AND PERSPECTIVE

In this review, we present a series of steps: control of hiPSC quality, expansion of hiPSCs, mass induction of CMs from hiPSCs, purification of hiPSC‐CMs, removal of residual undifferentiated hPSCs, evaluation of remaining undifferentiated hPSCs *in vitro* and *in vivo*, and evaluation of cardiac function after transplantation of hiPSC‐CMs.

### 8.1 Quality maintenance and mass preparation of hPSCs

First, we showed that it is necessary to maintain the quality of hPSCs, that is, to keep them in a state without significant genomic mutations; that is, it is necessary to show that the following genomic findings are extant as to rule out whether tumorigenicity could be present in the hiPSCs to be transplanted: no karyotype abnormality and no structural abnormality, including single‐nucleotide variants/single‐nucleotide polymorphs or copy number abnormalities of tumor‐related genes, which are referred to in the COSMIC census and Shibata list.^120^ It is necessary to induce large amounts of hPSCs without mutating tumor‐related genes, and so far, we found that tryptophan, an amino acid, proliferates hPSCs with high efficiency without causing genetic abnormalities. Furthermore, the addition of tryptophan to the culture medium is inexpensive, making mass maintenance possible. In addition, to realize medical treatment that provides uniform quality of treatment to patients with hiPSC products, it is important to develop a simple and inexpensive method to control the quality of hiPSCs and their evaluation method. In particular, since mass culture is necessary to achieve hiPSC‐based cell therapy, it is desirable to establish a method to evaluate the quality of hPSCs for mass culture.

### 8.2 Preparation of highly purified hPSC‐CMs for cell therapy

It is necessary to develop a technology that can stably and efficiently induce target cells from hPSCs. It is also necessary to develop a technology that enables non‐invasive detection of differentiation efficiency and eliminates residual undifferentiated hPSCs and non‐target proliferating cells. hiPSC‐CMs in 3D are easy to collect for quality evaluation, but it is difficult to collect cells in 2D. Therefore, an analysis method using culture supernatants would be very useful. In addition, if the possibility of residual undifferentiated hPSCs or non‐targeted proliferating cells is shown, a method for the specific removal of these cells should be established. To date, we successfully induced a large number of hPSC‐CMs in a multilayer culture plate, which enabled us to induce mass differentiation in 2D; however, many issues remain to be solved, such as the stability of differentiation and how to reduce the differences in properties between other lots as mentioned above. Although we have not reached that point yet, the purification of hPSC‐CMs using glucose‐ and glutamine‐depleted lactate‐supplemented medium, which utilizes the difference in metabolism between hPSC‐CMs and non‐CMs, has made it possible to obtain large numbers of differentiated CMs at low cost and with little effort.

### 8.3 Cardiac maturation for cell therapy

To build an ideal scalable manufacturing system for clinical‐grade therapy products, there have been various reports on the types of hiPSC‐CMs surviving in lactate medium, and it has been reported that hiPSC‐CMs survive in glucose‐ and glutamine‐depleted lactate‐supplemented medium are metabolically more mature.^57^, ^58^, ^121^ This is probably because the mitochondria are well developed to convert lactate to pyruvate and metabolize it in the mitochondrial TCA cycle. Although hiPSC‐CMs are known to be more immature than adult human cardiomyocytes, it has been reported that hiPSC‐CMs at 20 days of induction show better attachment for cardiac transplantation than those on day 8 or 30.^122^ This result implies that it is not necessary to prepare mature hiPSC‐CMs to the same level as host CMs before transplantation; rather, it is desirable to transplant moderately immature cardiomyocytes and let them mature *in vivo*. Furthermore, it has been reported that more mature CMs maintained in medium with FAs cannot survive well after freezing.^123^ Therefore, for transplantation of more mature hPSC‐CMs, it is necessary to find conditions that allow survival after freezing and good engraftment during transplantation.

### 8.4 Evaluation and elimination of residual undifferentiated hPSCs

Although there have been many reports on the removal of residual undifferentiated hPSCs, we have shown that inhibition of FA synthesis is very effective in the removal of hPSCs in CMs. This is because hPSCs are unable to synthesize FAs, which are components of the cell membrane during cell division. Although it may be difficult to remove residual undifferentiated hPSCs from differentiated cells that actively synthesize FAs, removal by metabolic differences between different cells may be a useful method because it does not involve genome editing or require the use of expensive antibodies. The evaluation of the persistence of residual undifferentiated hPSCs *in vitro* depends considerably on the detection limit of the instrument and the specificity and expression of the gene of interest. If a sensitive and inexpensive method is established, it will be possible to frequently assess the persistence of residual undifferentiated hPSCs during the course of an experiment, thereby enhancing safety. Finally, accurate assessment of the persistence of residual undifferentiated hPSCs after transplantation is still highly dependent on sensitive detection methods and the specificity of hPSC expression. At the same time, simplicity and cost must be considered so that the grafts can be monitored for a long time after transplantation.

## AUTHOR CONTRIBUTIONS

Y.M and S. T wrote the original manuscript. Y. M, Y. K, K. F, and S. T reviewed and edited the manuscript. Y. M and S. T acquired funding. All authors have read and agreed to the published version of the manuscript.

## CONFLICT OF INTEREST

K.F. is a CEO of Heartseed, Inc. S.T. is an advisor of Heartseed, Inc. S.T. and K.F. own equity in Heartseed, Inc. The remaining authors declare no conflicts of interest.

## Data Availability

Data sharing is not applicable to this article as no data sets were generated or analyzed during the current study.
